# *“Every day they keep adding new tools but they don’t take any away”*: Producing indicators for intermittent preventive treatment for malaria in pregnancy (IPTp) from routine data in Kenya

**DOI:** 10.1371/journal.pone.0189699

**Published:** 2018-01-03

**Authors:** George Okello, Rene Gerrets, Scholastica Zakayo, Sassy Molyneux, Caroline Jones

**Affiliations:** 1 KEMRI-Wellcome Trust Research Programme, Nairobi, Kenya; 2 Department of Anthropology, University of Amsterdam, Amsterdam, Netherlands; 3 Centre for Tropical Medicine and Global Health, University of Oxford, Oxford, United Kingdom; Centro de Pesquisas Rene Rachou, BRAZIL

## Abstract

**Background:**

Intermittent preventive treatment for malaria in pregnancy (IPTp) is part of a multi-pronged strategy aimed at preventing malaria in pregnancy in areas of moderate to high transmission in sub-Saharan Africa. Despite being formally adopted as a malaria prevention policy over a decade ago, IPTp coverage has remained low. Recent demands for action have incorporated calls to strengthen IPTp monitoring and evaluation systems, including the use of routine data, to measure coverage, track implementation and identify roadblocks to improving uptake. Concerns about the quality of malaria indicators reported through routine information systems are well recognized, but there are few data on the realities of IPTp recording practices in frontline facilities or their entry into District Health Information Software (DHIS2).

**Methods:**

Drawing on fieldwork conducted in two malaria endemic sub-counties in Kenya, we explore how local adaptations and innovations employed by health workers and sub-country managers to cope with a range of health system constraints, shape recording practices and in turn, the measurement of IPTp. Data were collected through observations, interviews, and document reviews. Data analysis and interpretation was guided by thematic analysis approach.

**Results:**

Measurement of IPTp was undermined by health system constraints such as stock-out of drugs and human resource shortages. Coping strategies adopted by health workers to address these challenges ensured continuity in service delivery and IPTp data generation but had variable consequences on IPTp data quality. Unclear recording and reporting instructions also led to lack of standardization in IPTp data generation. The use of redundant tools created significant data burdens which undermined service delivery in general.

**Conclusions:**

There is need to integrate monthly reporting forms so as to remove redundancies which exacerbates workload for health workers and disrupts service delivery. Similarly, data collection instructions in registers and reporting forms need to be clarified to standardize IPTp data generation across health facilities. There is also need to address broader contextual factors such as stock-out of commodities and human resource shortages which undermine IPTp data generation process.

## Introduction

Malaria infections during pregnancy are a major public health problem with serious consequences for the health of the mother, her foetus, and the new-born child [1]. In 2003, the WHO recommended the use of intermittent preventive treatment for malaria in pregnancy (IPTp) as one the strategies for the prevention of malaria in pregnant women living in areas of moderate to high transmission in sub-Saharan Africa [2]. IPTp involves administering preventive doses of sulfadoxine/pyrimethamine (SP) to pregnant women during their pregnancy. The 2003 guidelines recommended that IPTp was administered at least twice before delivery [2] but in 2012 these guidelines were updated, increasing the suggested frequency at which IPTp should be delivered to at least three times during pregnancy with the last dose being administered up to the time of delivery [3].

In sub-Saharan Africa, the proportion of pregnant women who receive at least three doses of IPTp has remained low [4] and the need to scale up IPTp alongside other malaria in pregnancy interventions has been recognized [5, 6]. Recent systematic reviews indicate that a combination of individual, structural, cultural, and wider health system factors constrain the delivery of this intervention [7, 8]. The latest ‘Global Call to Action’ on IPTp recommends that countries should use routine health information systems to monitor IPTp implementation, measure coverage, and identify barriers to successful implementation [6]. Where such data exist, they can be used to assess uptake of IPTp among pregnant women [9, 10].

Many countries in sub-Saharan Africa are currently using the District Health Information Software (DHIS2), a web-based health information system for the collation and reporting of routine health and management data [11]. Generating routine health indicators relies on practices of measurement and counting which remain problematic in many low-income countries [12–15]. Specifically, the production of IPTp indicators requires that health care providers accurately record their IPTp dispensing practices and that these records are subsequently reported and correctly transcribed into the DHIS2. While the weakness of routine health information systems in low resource settings is widely recognised, few data are available on the realities of collecting and reporting IPTp data at frontline health facilities [16, 17]. If DHIS2 data are to be used to generate indicators for measuring the implementation and coverage of IPTp, a better understanding of factors influencing indicator production and how these may undermine data quality is required. In this paper, we draw on empirical data collected as part of a broader study investigating how malaria indicators are produced at the local level in Kenya to describe how IPTp data are collected and reported through the DHIS2. Specifically, we describe the process and practice of IPTp data collection and reporting at health facilities and explore the factors influencing these practices; and critically assess the implications for the use of real time data in monitoring IPTp implementation and coverage.

## Methods

### Study setting

Kenya’s new constitution passed in August 2010 introduced a devolved system of government that, in 2013, transferred health service delivery functions from the central government to 47 semi-autonomous government units known as counties. The national government provides technical support and the policy framework but county governments are responsible for IPTp implementation. Counties are further divided into smaller administrative units known as sub-counties (previously districts). It is at the sub-county level that the routine data generated in health facilities are entered into the DHIS2. In line with WHO updated policy on IPTp, the current national malaria strategy aims to ensure that at least 80% of pregnant women living in 14 malaria endemic counties located in the coast and lake regions receive a minimum of three doses of IPTp, provided as directly observed therapy (DOT) in antenatal clinics [18]. According to the 2015 Malaria Indicator Survey findings, between 97–98% of pregnant women in these two regions received antenatal care from a skilled provider [19]. However, only 35% to 43% of these women received at least three doses of IPTp [19].

This study was conducted in two malaria endemic counties located in the coast and lake regions of Kenya. These two counties are among the 14 malaria endemic counties where core malaria prevention, diagnosis and treatment interventions have been scaled up over past decade [18]. In 2015, malaria prevalence was 27% and 8% in the lake and coast endemic regions respectively [19]. In each county, following discussions with the county managers we purposively selected one sub-county in which to study the process of data entry into the DHIS2. From each sub-county, two frontline health facilities (a dispensary and a health centre) were selected as sites for the conduct of an in-depth study of the production of routine malaria data. The selection of the four health facilities was informed by various considerations including, existing relationships, accessibility, and availability of a working laboratory. The study design was based on an ethnographic approach which allows for an in-depth exploration of a phenomenon in a small purposively selected sample. The results from such a study are restricted in their statistical generalizability but have the potential for the generation of knowledge that is transferable to other similar settings [20].

### Data collection and analysis

The ethnographic approach adopted for this study involved the use of a range of data collection methods including: observations of data recording, collation, reporting and management practices; informal conversation; and interviews with health workers involved in the process. Observations were conducted by GO and SZ between July 2014 and December 2015. To understand IPTp data recording practices, antenatal care registers in all four facilities were retrospectively reviewed. At the sub-county level, observations involved spending time at the health records office and taking part in data entry into the DHIS2 as well as participating in sub-county wide malaria monitoring and evaluation activities. Formal interviews (n = 22) were conducted with frontline staff and the sub-county managers who were responsible for collecting, collating, and reporting routine health data. To understand the national policy context of malaria indicator data generation, key informant interviews (n = 5) were also conducted with national managers who were well versed in malaria monitoring and evaluation activities and the routine health management and information system.

Preliminary findings were discussed during four feedback meetings that were held with a group of health workers drawn from other facilities in the two sub-counties (n = 35) and their managers (n = 17). All interviews and meetings were conducted in both English and Kiswahili and took place in locations that were convenient to participants. Where consent was provided, interviews/meeting proceedings were audio-recorded and subsequently transcribed and translated. Secondary data to supplement primary data was obtained through document review. Interview transcripts and field notes were imported to Nvivo 10 for data management and analysis. Data analysis was guided by the thematic content analysis approach [21].

### Ethical consideration

This study was approved by the Kenya Medical Research Institute (KEMRI) Scientific and Ethics Review Unit (SSC 2772). Permission to conduct the research was also obtained from the County Departments of Health and the sub-county health management offices. Prior to fieldwork, meetings were held with health facility managers and health workers to explain to them the nature and purpose of the study. Verbal consent was obtained for observations and written consent for all formal interviews. Permission to publish this paper was obtained from the director, KEMRI.

## Results

### Describing the four facilities

The four facilities (referred to in this paper as facility A, B, C and D) provided similar curative, preventive and promotive services but differed in staffing and workload (Table 1).

**Table 1:** Facility characteristics.

	Facility A	Facility B	Facility C	Facility D
Classification	Health centre	Health centre	Dispensary	Dispensary
**Staffing**				
Clinical Officers	2	1^1^	1^1^	0
Nursing Officers	4	3	4	2
Laboratory Technologists	3	1	1	1
Support staff (e.g. data clerks; and dispensers)	7	2	3	3
Others (e.g. records officers)	2^1^	4^1^	4^1^	1^1^
**Monthly workload on selected indicators**^2^				
Antenatal care (ANC) attendance	328	67	91	70
Number of women who receive 1 dose of IPTp	74	15	16	1
Number of women who receive 2 doses of IPTp	94	13	16	2

^1^Staff employed by local NGOs to provide HIV/AIDS care and treatment services.

^2^Data obtained from the DHIS2 and represent average monthly workload in 2015.

**Fig 1:**
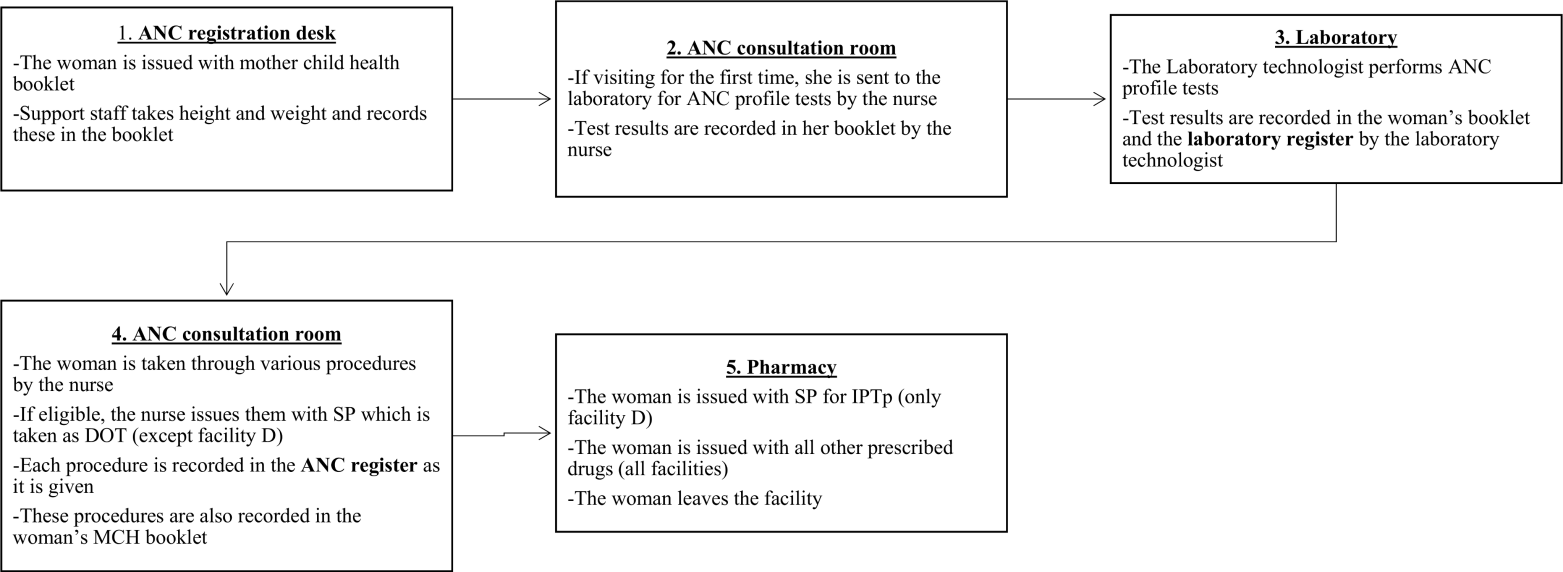
The antenatal care visit process.

Facility A was the busiest owing to its location in a busy urban centre. It also had the highest number of staff. Facility D administered the least number of IPTp doses in a month. This low level of IPTp dispensing was attributed to a prolonged stock of out SP that lasted for close to 8 months in this facility. The remaining three facilities also experienced intermittent stock-outs of SP which was a nation-wide problem at the time of this study [22]. Antenatal care (ANC) services were primarily provided by nurses in all four facilities although clinical officers in facility B & C occasionally assisted nurses in the provision of these services. Generally, sharing of roles and informal task shifting was common across a range of staff working in all four facilities. ANC services were provided on a daily basis in facilities A, B and C and once a week in facility D due to shortage of staff. The ANC visit process is summarized in Fig 1.

### IPTp data collected and reported at the health facility

At the time of this study, the specified tool for capturing IPTp data at facility level was the standard Ministry of Health Antenatal Care register which captured a range of information related to a woman’s pregnancy. The ANC register had one column per page for recording IPTp. The attending health worker was expected to record the dose of IPTp given as it was given. At the bottom of each page of the register, the attending health worker was required to summarize the total number of pregnant women who received one dose of IPTp (*No*. *given IPTp1*) and those who received more than two doses of IPTp (*No*. *given IPTp2+)*. At the end of each month, IPTp data from the ANC register were tallied and reported in four separate monthly reporting forms (Table 2).

**Table 2:** IPTp reporting forms.

Reporting form	IPTp indicator reported	Where submitted
1. National Integrated Summary Report	• Number of clients given IPTp1 • Number of clients given IPTp2	Sub-county health records office
2. Annual Work Plan Report	• Total number of women given IPTp2 in epidemic and endemic districts	
3. Service Delivery Report	• Total number of pregnant women given IPTp2	
4. Malaria Commodities Form	• Total number of pregnant women receiving IPTp	Sub-county pharmacy office

These four completed reporting forms, along with other monthly reports were supposed to be submitted to sub-county offices by the 5^th^ of every month for entry into the DHIS2 by the 15^th^ of every month. Data entry into the DHIS2 in both sub-counties was primarily undertaken by volunteers or interns, in some cases using personal or borrowed laptops, with very minimal supervision from sub-county managers.

### Factors influencing IPTp recording and reporting

A number of factors were found to influence IPTp data recording and subsequent reporting in the four facilities. These can be grouped as factors relating to: *IPTp administration and dispensing practices; data collection and reporting tools; indicators* and *reporting requirements*.

#### a) IPTp administration and dispensing practices: Informal task shifting

The national policy for IPTp administration is directly observed therapy (DOT) with the health worker recording the observed dose in the ANC register as it is given [23]. In facilities A, B and C, whenever SP tablets were in stock, health workers generally adhered to the DOT policy and recorded the dose in the ANC register and the woman’s booklet while the woman was still in the ANC room. There were a few instances when health workers issued SP tablets to pregnant women and instructed them to take these at home. These were recorded in the ANC register as IPTp doses administered although they were not given as DOT. In facility D, the task of IPTp administration had been shifted to the pharmacy. Women were prescribed SP by a nurse in the ANC consultation room and instructed to collect the drug from the pharmacy. The IPTp dose was recorded by the nurse in the ANC register and the woman’s booklet as if it had been dispensed by DOT. In some cases, the dispenser (a support staff) in the pharmacy issued SP as DOT. However, when the queue of patients waiting to be served in the pharmacy was long, the dispenser issued SP tablets to women to take at home. One of the health workers explained that their decision to shift SP administration to the pharmacy was to reduce the workload on the nurses and that it made sense to do this because other drugs requiring DOT were prescribed and dispensed in this way.

“I: So why was it [SP] prescribed this side [ANC clinic] but administered in the other room [pharmacy]?

R: To reduce the workload…

I: Is it not supposed to be administered as DOT?

R: Yes. We assumed that if Artemether Lumefantrine is administered as DOT in the pharmacy, then even SP can be administered as DOT in the pharmacy”.

It was only rarely that any record was made in the pharmacy of IPTp dispensing practices. It was therefore not possible to verify if IPTp prescribed by a nurse had been administered by the dispenser. Nonetheless, this practice may contribute to over-reporting of this indicator if women do not take the drug at home as instructed.

In all four facilities, support staff played a crucial role in service delivery and the health data collection process. Although their involvement in non-clinical service delivery freed health workers to concentrate on the delivery of services requiring clinical skills, there were instances where support staff were blamed for poor quality data. For example, in a review of the ANC registers in facility A, it was noted that between January and February of 2015, IPTp doses administered were simply marked as ‘Y’ or ‘1’ in the ANC register which made it impossible to identify the dose of IPTp given to a woman. Nonetheless, the facility still produced reports on IPTp1 and IPTp2, although none of the health workers in this facility could explain the source of this data. Conversations with staff working in the ANC clinic about this anomaly revealed that around this period, the ANC register was filled by support staff who had not been properly trained in data recording.

#### b) Data collection tools: Unclear recording instructions

The instructions on how IPTp should be recorded that are provided inside the front cover of the ANC register instructed health workers to: ‘*Write the dose which has been given or NO if not given*. *If the woman is not eligible record ‘NA’ for not applicable’*. Whenever IPTp was administered, with a few exceptions, recording practices were largely uniform across facilities and health workers (i.e. IPTp recorded as 1, 2, 3… up to 7), but there were marked variation in recording practices when IPTp was not given to a pregnant woman. According to national guidelines, there were three main instances when IPTp was not supposed to be administered: i) if the woman was in the first trimester of pregnancy; ii) if the woman was on cotrimoxazole (CTX) prophylaxis for the prevention of opportunistic infections in HIV/AIDS infected patients, making them ineligible for SP; and iii) if the mother had been given a high dose of folic acid [23]. In addition to these three scenarios, there were additional instances reported by health workers when SP was not administered to a pregnant woman such as if the woman was allergic to SP, or if SP was out of stock. Instructions in the register were unclear regarding how each of these events was supposed to be recorded, which created confusions leading to variability in recording practices as discussed during one of the feedback meetings.

Moderator: So when do you write not applicable?

Participant 5: In fact, I don’t write not applicable. It’s either a NO or 1st, 2nd, 3rd. NO means not given. So the reasons could be HIV, it (SP) is out of stock. She is allergic…

Participant 3: I write NO… NO…

Moderator: For everything?

Participant 3: Yes

Participant 4: No includes everything

Participant 1: That is where the problem is. Because everyone understands things differently. When someone writes a NO, the NO can mean other things”

Not being able to distinguish in the daily ANC register the reason why a dose of SP had not been given to a pregnant woman was clearly an issue for the health workers across all four facilities as they had developed a series of their own annotations (often unique to each facility) to provide more specific information on why IPTp had not been issued. For instance, in facility C, to indicate that a woman was on cotrimoxazole prophylaxis for HIV which disqualified her from getting IPTp, they recorded ‘CTX’ in the IPTp column even though this information was also collected in a separate column in the register. Staff explained that this made it easier for them to identify women on cotrimoxazole prophylaxis in the future, a practice also reported by one of the health workers during the feedback meeting.

“If the mother is HIV positive, I normally just write ‘CTX’ so that somebody can know that this mother is on Septrin [cotrimoxazole prophylaxis] so cannot use Fansidar”.

Stock-outs of SP, a nation-wide problem at the time of this study [22], resulted in variations in IPTp recording practices. Generally, when facilities ran out of SP, health workers gave pregnant women a prescription, and asked them to purchase the drug at a local pharmacy. These events were variably labelled in the ANC register. For example, in facility C, health workers recorded ‘*to buy*’ in the ANC register. In Facility D, the nurse prescribed the drug and urged women to purchase it in local pharmacies. This was recorded as ‘N’ (not issued) in the register. In facilities A & B, SP stock-out information was marked as ‘O/S’ in the IPTp column in the register.

*“We write OS [out of stock] because somebody might come to check the book to see why this woman never received SP*. *So you write OS because you know that this lady was supposed to get SP but you didn’t issue because it was out of stock*.*”* Health worker, Facility A

#### c) Inconsistent indicators

Data extraction from the ANC register into the various monthly reporting forms was complicated by the different wording used in the various forms (Table 2). Transferring information on the number of women who received one dose of IPTp from the ANC register into the Integrated Monthly reporting form was straight forward as the ANC register page summaries of ‘*No*. *given IPTp1’* translated directly into the Integrated Monthly reporting form indicator ‘*No*. *clients given IPTp 1*^*st*^ *dose’*. However, the summary in the ANC register for two or more doses of IPT read: *No*. *given IPT2*+, while the indicator in Integrated Monthly reporting form, Annual Work Plan reporting form and Service Delivery reporting forms specifically required data on *‘number of pregnant women who received the second dose of SP for IPTp (IPTp2)’*. This inconsistency between page summary data and monthly reporting requirement coupled with unclear guidelines on IPTp implementation following the change in IPTp policy created confusions which led health workers to report IPTp2+ in place of IPTp2, i.e. all women receiving two to seven doses of IPTp were counted and reported as IPTp2. This over-reporting of the IPTp2 indicator was a well-recognized problem in Kenya [22] and resulted in corrective actions such as refresher training for health workers, and demands for health workers to recount and resubmit IPTp 1 & 2 data for the three preceding years in one of the two sub-counties for correction in the DHIS2 [24]. Despite these actions, at the time of the study these inconsistencies were still causing confusion in the participating health facilities.

#### d) Reporting burdens

There were constant complaints from health workers and their managers who observed that most of the data collected and reported routinely were duplicated across various report forms. They were concerned that much of this repetition was unnecessary and was increasing their workload and undermining their capacity to deliver services.

*“My concern is the issue of duplication of data*. *I don’t know but I think at the national level*, *they need to integrate some of these tools*. *It’s an issue because the health workers are being overwhelmed by the many tools…?”* Sub-county Manager

For instance, the *number of pregnant women who received IPTp2* was captured in three different reporting forms. Although the label attached to the IPTp2 indicator differed slightly across the three forms, in each case the figure reported was extracted from the ANC register and, therefore was the same in all three forms (Table 3).

**Table 3:** Facility B IPTp2 DHIS data 2015.

Report	Indicator name	Jan	Feb	Mar	Apr
Annual Work Plan report	Number of pregnant women receiving IPT2 in endemic and epidemic districts	12	34	25	13
Service delivery report	Number of pregnant women receiving IPT2	12	34	25	13
Integrated Monthly report	Number of clients given IPT (2nd dose)	12	34	25	13

These three reporting forms were said by the health workers to contain the most number of duplicated indicators. Observations of these reporting forms suggested that there were several indicators that were duplicated (including non-malaria indicators). Although Annual Work Plan and Service Delivery reporting forms were manually completed at the health facility level, several data fields in these forms were not manually keyed into the DHIS2 in the sub-county health records offices. Instead, data fields in these two forms were auto-completed by the DHIS2 software using data recorded in other monthly reporting forms hence leading to concerns about the rationale for completing these paper forms at the health facility.

Document reviews and interviews with managers at the national and sub-county level revealed that indicators in the Service Delivery reporting form report were used to monitor the goals of the health sector strategic plan which had been in place prior to 2014 [25]. However, this had been replaced with the new health sector strategic plan [26]. The Annual Work Plan report was introduced as a replacement of Service Delivery report which should have been withdrawn from use. Sub-county managers were aware of this but they explained that since they had not received official communication from the national government, they could not withdraw the MOH 105 from use in their facilities.

*“Service delivery is actually supposed to cease*. *We are supposed to stop it*, *but you know we have not gotten clear communication from national*. *So at my level I can’t communicate”* Sub-county Manager

Throughout the study period, both Service Delivery & Annual Work Plan reporting forms remained in use. This led to duplication in reporting of many indicators (not just IPTp and malaria) retrieved from many different registers. Manually filling these reporting forms was a tedious process as, for instance, Service Delivery reporting form contained about 63 fields, Annual Work Plan reporting form contained 71 data fields and Integrated Monthly reporting form contained over 300 data fields. In addition, during the study period there was a shortage of standard reporting tools containing automatic carbon copying, resulting in health workers manually duplicating data in these three forms (original copy forwarded to the sub-county records office and duplicate copy retained in the facility for record keeping). These reporting processes took a huge amount of time, in some cases, disrupting normal service delivery when health workers were under pressure to beat reporting deadlines.

Many of the health workers and their managers pointed to the need for integration of existing tools and indicators.

“I think the tools needs to be integrated. Every day they keep adding new tools but they don’t take any away. When you look at the new tools that they add, they ask you to report same things that you have been reporting in the other forms. Let's say [HIV/AIDS] or even malaria reporting forms. Whatever you report on this form is what you report on the other form. So the [sub-county health records officer] will call you to ask you why data in [HIV/AIDS report] and [Integrated Monthly report] are inconsistent. So you ask yourself why they asked you to fill the same data in two different forms which are all sent to the same place”

## Discussion

There is currently great interest in the potential for using ‘real time’ malaria data to measure coverage of key malaria interventions such as IPTp and inform local decision-making [9, 10, 27]. However, there is also widespread acknowledgement of the deficiencies of much of the data produced through routine health information systems [28, 29]. Several interventions have been developed and implemented in an attempt to improve the quality of these processes and their outputs [30–32] and the computerisation of many district health information systems in sub-Saharan Africa was, in part, fuelled by the prospect of improved health data demand and use [33–36]. However, most of these interventions have focused on the technical requirements for data quality and few have taken account of the broader context within which indicators are produced. This study investigated the process of IPTp data generation for entry into the DHIS2 to identify how on the ground realities affect the measurement of IPTp coverage.

The results demonstrate that measuring IPTp at the frontline is shaped by the wider health system challenges which have been identified as some of the major constraints in the delivery of IPTp [8]. The adaptations and innovations employed by health workers to respond to these challenges subsequently influence the quality of the indicators produced in the DHIS2. As such, the generation of reliable indicators from routine data is dependent on the adequate function of all elements of the health system (human resources, medicines and technologies; governance; information; financing; and service delivery) mediated by the needs, motivations and relationship of the people working in the system; not just the sub-system of health information [37].

Informal task-shifting, adopted as a coping mechanism to address staff shortages ensured continuity in service delivery but undermined both the provision of IPTp as per the policy guidelines (DOT) and contributed to data quality issues (recording of doses as given when not observed and inaccurate recording by untrained staff). These types of practice were observed in all four facilities involved in this study and have been cited as a cause for concern in a recent national data quality assessment report [38]. Adding to this problem in Kenya is the recent decision by the national malaria control programme to drop malaria in pregnancy indicators from the list of malaria indicators which are routinely audited, as well as a lack of funds for support supervision which has prevented sub-county managers from visiting facilities for routine supervision where some of these inconsistencies may be revealed [39]. Task shifting has been promoted in sub-Saharan Africa as a possible strategy for addressing well recognised financial constraints and staffing challenges in the region, and improving service delivery [40]. Our observations suggest that such strategies would require the provision of training opportunities, adequate support supervision and effective regulatory frameworks, to ensure both effective service delivery and adequate data recording and reporting practices [41].

Stock-outs of SP was one of a variety of reasons that IPTp might not be given to a pregnant woman attending the ANC. However, the ANC register in which the administration of IPTp is captured does not have an appropriate code for reporting this explanation. Front line health workers were concerned that their inability to accurately record the reason for not providing SP might have consequences for re-stocking. To cope with these reporting constraints the health workers in all four facilities had developed a series of their own annotations (often unique to each facility) to provide more specific information on why IPTp had not been issued; suggesting a real concern about why IPTp has not been administered. If these data could be systematically captured they may help in understanding the IPTp administration practices which undermine its effective implementation and contribute to low coverage. Furthermore, existing data quality audits tools tend to focus more on what is counted and reported (IPTp doses administered) as opposed to what is not reported [42], therefore overlooking some of the data recording practices observed in this study.

The burden of reporting in the health facilities was huge with, for example, the IPTp information recorded in one register, transcribed into multiple paper reporting forms and subsequently entered into three separate electronic forms in a central database at the sub-county. Despite the workload, none of the indicators collected and reported at the time of this study provided useful data for tracking the country’s progress towards the targeted 80% coverage with a minimum of three doses of IPTp by 2018. While the national malaria strategy had been revised to reflect updated policy, indicators in the national malaria monitoring and evaluation plan were yet to be revised to reflect this policy update. Capturing the additional data required to monitor the number of pregnant women who receive a minimum of three doses of IPTp would only require a very minor modification of the Integrated Monthly reporting form (paper and electronic in the DHIS2). However, caution is required to ensure that as a new indicator is added the old indicator is removed to avoid adding to the growing pile of redundant information. As has been reported elsewhere, there is an urgent need to review existing data collection and reporting tools to remove duplications and reduce data burdens associated with internal and external demands for accountability which is a major issue for frontline health staff [43].

Poor communication from the national level to counties and sub-counties of changes in recording and reporting requirements associated with the updating of the Kenyan health sector strategic objectives has added to the unnecessary reporting burdens at front line health facilities (e.g. the filling of the Annual Work Plan forms). The lack of clarity around the rationale for the presence of the Annual Work Plan form in frontline health facilities suggests that health information system accountability practices are primarily top-down. There seems to be little opportunity for providers at the front line to provide feedback and input to the reporting system and the dialogue between managers at sub-county level and national level decision makers appears to be limited. The obvious disconnect between the data recorded in the health facility registers (IPTp 1–7) and the data extracted from this register and subsequently reported through the Integrated Monthly reporting form (only spaces for IPTp1 & IPTp2) further points to a lack of coordination among key stakeholders at national level; and inconsistencies in IPTp implementation policy and indicators for monitoring and evaluation were also evident in various national guidelines and training manuals [23, 44, 45]. Such national level challenges have been identified as one of the potential barriers to the effective implementation and evaluation of IPTp across a range of countries in Africa [46]. These findings suggest that addressing the challenges of the poor quality of data produced at front line health facilities will require a focus that moves beyond recording practices and the facility itself. It will also need to incorporate an understanding of the national and local context within which the information is produced. Health worker actions are influenced by the systems within which they are practiced and interventions to change actions and improve IPTp measurement need to take account of the broader challenges that they face in providing quality health care and recording their practices.

One of the limitations of this study is that it was based on a small sample of health facilities and sub-counties that were purposively sampled. As such, these findings are not statistically generalizable. However, the ethnographic approach adopted in this study allowed an in-depth understanding of key issues around IPTp data generation practices and processes that are potentially transferrable to similar resource constrained settings. The use of multiple sources of data collection and validation of results through feedback meetings improved analytical generalizability of these findings. Another limitation of this study was our inability to conduct more interviews with county and national levels managers. Specifically, interviewing representatives from a diverse range of managers at these levels as well as other stakeholders involved in malaria control in Kenya would have added depth to the findings of this study and its analytical generalizability.

## Conclusion

There is need to modify existing monthly reporting forms to collect data on the number of pregnant women who receive at least three doses of IPTp as recommended in the current strategy. In addition, there is need to integrate monthly reporting forms to remove redundancies which exacerbates the workload for health workers and disrupts service delivery. There is also need to have clear instructions in the ANC register so as to address some of the variations in IPTp data recording practices that were observed in this study. This study has also demonstrated that the challenges in producing IPTp indicators through the DHIS2 are embedded within the broader challenges faced by the health system. As such, any intervention that seeks to improve IPTp data generation must look beyond IPTp or routine health information systems and address broader contextual factors that influence the process.
